# Fibrogenesis and Carcinogenesis in Nonalcoholic Steatohepatitis (NASH): Involvement of Matrix Metalloproteinases (MMPs) and Tissue Inhibitors of Metalloproteinase (TIMPs)

**DOI:** 10.3390/cancers6031220

**Published:** 2014-06-27

**Authors:** Isao Okazaki, Takuji Noro, Nobuhiro Tsutsui, Eigoro Yamanouchi, Hajime Kuroda, Masayuki Nakano, Hiroaki Yokomori, Yutaka Inagaki

**Affiliations:** 1 Department of Internal Medicine, Sanno Hospital, International University of Health and Welfare, Tokyo 107-0052, Japan; 2 Department of Internal Medicine, International University of Health and Welfare Hospital, Tochigi 329-2763, Japan; 3 Preventive Medical Center, International University of Health and Welfare Hospital, Tochigi 329-2763, Japan; 4 Department of Surgery, International University of Health and Welfare Hospital, Tochigi 329-2763, Japan; E-Mails: norotaku@gmail.com (T.N.); t-nobuhiro@iuhw.ac.jp (N.T.); 5 Department of Radiology, International University of Health and Welfare Hospital, Tochigi 329-2763, Japan; E-Mail: yamanouchi@iuhw.ac.jp; 6 Department of Pathology, International University of Health and Welfare Hospital, Tochigi 329-2763, Japan; E-Mail: hajimek@iuhw.ac.jp; 7 Department of Pathology, Ofuna Chuo Hospital, Kanagawa 247-0056, Japan; E-Mail: masayuki-nakano@ofunachuohp.net; 8 Department of Internal Medicine, Kitasato University Medical Center, Saitama 364-8501, Japan; E-Mail: yokomori@insti.kitasato-u.ac.jp; 9 Department of Regenerative Medicine, Tokai University School of Medicine and Institute of Medical Sciences, Isehara 259-1193, Japan; E-Mail: yutakai@is.icc.u-tokai.ac.jp

**Keywords:** hepatocellular carcinoma (HCC), nonalcoholic steatohepatitis (NASH), nonalcoholic fatty liver disease (NAFLD), matrix metalloproteinase (MMP), tissue inhibitor of metalloproteinase (TIMP), cancer invasion, cancer metastasis, bone marrow-derived stem cell, hepatic progenitor cell (HPC), cancer stem cell

## Abstract

Nonalcoholic steatohepatitis (NASH) is emerging worldwide because life-styles have changed to include much over-eating and less physical activity. The clinical and pathophysiological features of NASH are very different from those of HBV- and HCV-chronic liver diseases. The prognosis of NASH is worse among those with nonalcoholic fatty liver diseases (NAFLD), and some NASH patients show HCC with or without cirrhosis. In the present review we discuss fibrogenesis and the relationship between fibrosis and HCC occurrence in NASH to clarify the role of MMPs and TIMPs in both mechanisms. Previously we proposed MMP and TIMP expression in the multi-step occurrence of HCC from the literature based on viral-derived HCC. We introduce again these expressions during hepatocarcinogenesis and compare them to those in NASH-derived HCC, although the relationship with hepatic stem/progenitor cells (HPCs) invasion remains unknown. Signal transduction of MMPs and TIMPs is also discussed because it is valuable for the prevention and treatment of NASH and NASH-derived HCC.

## 1. Introduction

Simple hepatic steatosis, or fatty liver, is often seen in patients with obesity, diabetes mellitus, dyslipidemia and metabolic syndrome in the developed and developing countries (reviewed in [1,2,3,4,5]). Among them, the patients who never drink alcohol, or drink less than 70 g/week for women and 140 g/week for men are defined as nonalcoholic fatty liver disease (NAFLD) if the patients do not have any drug history, negative HBV- or HCV-related markers or negative markers for autoimmune liver diseases, and fatty liver is observed by hepatic echography, CT or MRI (reviewed in [1,2,3,4,5,6]). NAFLD is seen in 9% to 37% of the general population, and is currently emerging in both developed and developing countries due to changing life-styles (reviewed in [1,2,3,4,5,6]).

Nonalcoholic hepatitis (NASH) reported by Ludwig *et al.* in 1980 [7], has been clarified to be a severe form of NAFLD; 13% to 31% cases of NAFLD progress to NASH (reviewed in [4,5]). NASH is very similar to alcoholic hepatitis (ASH) in pathology (reviewed in [1,2,3,4,5,6,7]). Its prognosis is somehow better than that of ASH (reviewed in [1]), but 9% to 20% of NASH patients usually progress to liver cirrhosis (reviewed in [3,4,5,6,8]). NASH is seen in 2% to 5% of the general population (reviewed in [4,5]). NAFLD include a large spectrum of chronic liver diseases from simple hepatic steatosis or fatty liver, through NASH, to cirrhosis post-NASH. As NASH progresses to cirrhosis, steatosis progressively disappears with the development of fibrosis to cirrhosis, a phenomenon known as “burn-out NASH” [8], and such cases may present as cryptogenic cirrhosis ([9], reviewed in [1,2,3,4,5,6,7,8]). As it is difficult to discriminate NASH among NAFLDs, scoring of histological findings by liver biopsy has been developed [10].

Some patients with NASH show hepatocellular carcinoma (HCC) with or without liver cirrhosis [11]. Hepatocellular carcinoma (HCC) ranks third in cancer mortality and annual deaths number over 600,000 [12]. Studies have shown that HCCs are due mainly to hepatitis B virus (HBV) infection (50% to 80%) and hepatitis C virus (HCV) infection (10% to 25%) (reviewed in [13]). Geographical endemic infection in the World varies: in China nearly 99% HCC are reported to be HBV-related, while 12% is HCV-related in another report; in Gambia HBV-related HCC is 61%, while HCV-related HCC is 19%; in Japan HBV-related HCC is 15% and HCV-related HCC 61%; in the USA HBV-related HCC is 16% and HCV-related 36% (reviewed in [8,13]). HCC patients with HBV (with HIV) or HCV have shown HBV-related or HCV-related chronic hepatitis and/or liver cirrhosis prior to development of HCC (reviewed in [13,14]). In the USA 22% of HCC cases is associated with alcohol-induced liver disease and more than 40% associated with diabetes, NAFLD and NASH (reviewed in [13]). In Japan 15% of HCC is associated with diabetes, metabolic syndrome, NAFLD and NASH (reviewed in [13]). In developed countries (Japan, USA, Europe) HCV-related HCC has decreased, but a new trend in HCC development is emerging with changes in environment and lifestyles, e.g., the growing burden of diabetes, metabolic syndrome and obesity (reviewed in [13]).

We have investigated fibrogenesis and fibrolysis in liver diseases ([14,15,16,17,18,19,20,21,22,23,24,25,26,27,28], reviewed in [29,30,31]) as well as the occurrence and stromal invasion of hepatocellular carcinoma (HCC) from the viewpoint of MMP science ([32,33,34], reviewed in [35]). The present review discusses the fibrogenesis of NASH, the relationship between fibrosis and HCC occurrence in NASH, and finally introduce past, present and future prospects of HCC carcinogenesis and its relation to MMPs and TIMPs for application to HCC treatment. 

## 2. Method for Selection of References

The papers listed in this review dated before 2000 were selected from review articles previously published by the present authors [29,30,31,35]. Those published after 2000 were chosen from PubMed. The keywords used in the search were as follows: NASH, fibrogenesis and MMPs; NASH, carcinogenesis and MMPs; hepatitis B or C virus-derived chronic hepatitis and MMPs. We selected those papers published after 2000 that we considered to be most important and appropriate. The purpose of this review is not to extract any conclusive research result as in a meta-analysis. We introduce basic and novel science reports focusing on fibrogenesis and carcinogenesis and their relation with MMPs and TIMPs for this review.

## 3. Pathophysiology of NASH

**Figure 1:**
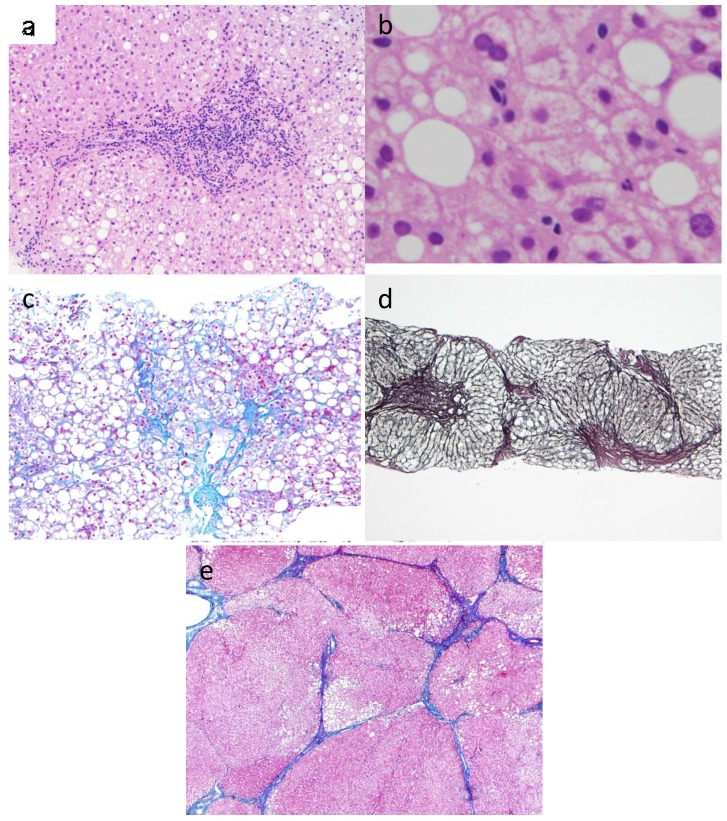
Histological features of the different steps of fibrosis in NASH. (**a**) NASH: Steatosis, hepatocyte balooning degeneration, and inflammatory cells composed predominantly of lymphocytes in the portal area (Hematoxylin-eosin stain, 200× magnification); (**b**) Mallory body in NASH showed staghorn pattern (Hematoxylin-eosin stain, 400× magnification); (**c**) NASH: Detail of wire-mesh fibrosis and steatosis (Azan stain. 100× magnification); (**d**) Bridging fibrosis: Portal-central fibrous septa linking portal tracts and central veins (Reticulin silver stain, 40× magnification); (**e**) NASH-derived cirrhosis: Larger nodules with thin fibrous septa and steatosis (Azan stain. 40× magnification).

NAFLD comprises the following three groups: simple steatosis or fatty liver (FL), few inflammatory lesions around the portal triad (FL-IN) and NASH [3]. In NASH, four histological features are observed, *i.e.*, steatosis, lobular inflammation, hepatocellular ballooning and fibrosis [3,5,6,7]. A NAFLD activity score (NAS) has been developed to classify NAFLD cases into “NASH”, “borderline” and “not NASH” [10]. Balooning and Mallory bodies (mitochondrial degeneration of hepatocytes) are considered to be necrotic processes in NASH [3,5]. These characteristic alterations followed by fat deposition in hepatocytes may cause more severe, chronic liver inflammatory lesions (Figure 1a,b). In the early stage of NASH slight fibrosis is seen around the tributaries of central veins. Fine fibrous bands are formed around hepatocytes in zone 3, known as “wire-meshed”, “pericellular” and “chicken-wire” fibrosis (Figure 1c). The second stage, “bridging fibrosis”, is the connecting fibrosis between zone 3 and the fibrosis around the portal area including arteries, veins and bile ducts (Figure 1d). The fibrosis progresses into the nodule, and circles the newly formed regenerative nodule, resulting in liver cirrhosis (Figure 1e).

These progressions are also seen in alcoholic fatty liver to alcoholic cirrhosis, but are very different from hepatitis B virus-derived chronic hepatitis to cirrhosis and hepatitis C virus-derived chronic hepatitis to cirrhosis. Pericellular fibrosis and/or perivenular fibrosis around the central vein tributaries in zone 3 is observed in the early stage of NASH, while the fibrosis around the portal or bile ducts is seen in chronic viral hepatitis [3,5].

The whole spectrum of NAFLD occurs mostly in patients with obesity (60% to 95%), type 2 diabetes mellitus (28% to 55%), and dyslipidemia (27% to 92%) [36]. In NASH, the first hit is the occurrence of NAFLD, and a second hit may cause more severe inflammation and fibrosis [37]. One of the second hits shows a pattern of fat distribution, the accumulation of visceral fat [38]. Moreover, abnormal glucose regulation causes increased risk for NASH [38]. Increase in BMI is a significant risk [39]. BMI and diabetes have also been found in cross-sectional studies to be associated with advanced fibrosis in patients with NASH [39]. TG accumulation in the liver is usually a result of *de novo* lipogenesis in increased free fatty acids derived from diet and peripheral lipolysis on adipocyte tissue. Among those suffering from obesity *de novo* lipogenesis is markedly increased, contributing to promote hepatic steatosis. These processes may be due to “insulin resistance,” *i.e.*, insulin inhibits glucose production and promotes lipogenesis. Hepatic insulin resistance was also caused by increased methylation of the gene promoter region in the catalytic subunit of protein phosphatase 2A [40]. PNPLA3 and other genes may be implicated in promoting TG deposition in the liver (reviewed in [1,2,41]).

## 4. Mechanism of Fibrogenesis in NASH

### 4.1. Fibrogenesis in the Liver: Cells Responsible for ECM Formation, Cytokines, Signal Transduction, Role of Bone Marrow (BM)-Derived Cells

The deposition of ECM in the liver is based on the balance of ECM formation and degradation. Hepatic stellate cells (HSCs; Ito cells; fat-storing cells) localized in the perisinusoidal space have been clarified to be the most important producer of ECM. HSCs have several unique components such as vitamin A-related components and lipids, and cytoskeletal markers (vimentin, desmin) in normal liver. HSCs transform into proliferative, fibrogenic and contractile myofibroblasts following liver injuries (reviewed in [42]). This phenotypical transdifferentiation of HSCs is known as activation of HSC transferred from quiescent HSC under a physiologically normal condition. The activated HSCs (myofibroblasts) lose retinoid storage fat and express unique cytoskeletal markers (α-SMA, GFAP, nestin), cytokine receptors (PDGF-R, TGFβ-R type I, II, and III, ET-R, EGF-R, VEGF-R), other receptors [integrin, DDRs (discoidin domain receptors), thrombin-R, mannose-6-phosphate-R, uPA-R], signaling components (raf and MAP kinase), and transcription factors (Sp1, NFκB, Z19/KLF6). HSCs produce very important cytokines, growth factors and inflammatory mediators (prostanoids, leukocyte mediators (M-CSF, MCP-1, PAF), acute phase components (α_2_-macroglobulin, IL-6), mitogens (HGF, EGF, PDGF, SCF, IGF-I, -II, αFGF), adhesion molecules (I-CAM-1, V-CAM-1, N-CAM), vasoactive mediators (ET-1, NO), fibrogenic cytokines (TGF-β1, -β2, β-3, CTGF), IL-10, C1NC), extracellular matrix such as collagen (type I, III, IV, V, VI, XIV) and proteoglycans (heparin, dermatan and chondroitin sulfates, perlecan, syndecan-1, biglycan, decorin), glycoproteins (cellular fibronectin, laminin, merosin, tenascin, nidogen/entactin, undulin, hyaluronic acid), proteases (MMP-1, -2, -3, -14) and protease inhibitors (TIMP-1, -2, PAI-1) (reviewed in [43,44]).

Among them, mitogens such as PDGF, TGFα and EGF stimulate mitosis in HSCs, creating an *autocrine* loop for cellular proliferation. Pinzani and Mara revealed that PDGF is the most potent mitogen for cultured HSC isolated from rat, mouse, or human liver, and sequential signal transmission followed by phosphatidylinositol 3-kinase (PI 3-K) activation is necessary for both mitogenesis and chemotaxisis induced by PDGF in human HSC cultures (reviewed in [45]). HGF causes hepatocyte mitosis followed by the activation of HSCs. The injured hepatocytes, the activated Kupffer cells, and the activated endothelial cells can produce and secrete cytokines which bind to the receptors of HSCs mentioned above, and HSCs are activated to become myofibroblasts in a *paracrine* manner followed by wound healing. DDRs may mediate interactions between stellate cells and the surrounding interstitial matrix during progressive liver injury (reviewed in [43,44]). The different phenotypes of HSCs in normal and injured liver are regulated as the result of interactions with neighboring cells through *paracrine* and *autocrine* pathways as well as the interactions between HSCs and changes in ECM. This process is called the “*initiation*” phase of HSC activation. The second phase of HSC activation is “*perpetuation*”, that is, myofibroblats proliferate, migrate to the site of the liver injury and produce excessive amounts of ECM resulting in scarring of the liver (reviewed in [43,44,45,46]).

The fate of the activated HSC is not only to progress to apoptosis but also to regress to quiescent HSC. Tsukamoto’s group has reported dynamic phenotype reversibility regarding this problem. Twenty years ago they found PPARγ to be a regulator for the activation of HSC, similar to adipogenic transdifferentiation (reviewed in [47]). Very recently they found that HSC-derived Delta-like Homology 1 (DLK1) protein activates HSC via epigenetic repression of the master adipogenic gene *Pparγ* in a manner dependent on canonical Wnt [48]. Up-regulation of DLK1 participates in liver regeneration after partial hepatectomy followed by hepatocyte proliferation and liver growth. They also demonstrated that DLK1 knockdown reverses activated HSCs to fat-storing quiescent cells via epigenetic derepression of *Pparγ*. Moreover, their group [49] showed that myofibroblasts revert to an inactive phenotype in the recovery phase from liver fibrosis. However, inactivated HSCs are sensitive to the stimulator to transdifferentiate again to the active HSC. The cell markers of sinusoidal HSCs have been reported to be different to those of portal myofibroblasts. Vimentin, desmin, HSP47 and αSMA are expressed by both HSCs and portal myofibroblasts. CD95L, α2-macroglobulin, p100, reelin, fastin [50] and cytoglobin [51] are expressed in HSC, NCAM in human HSCs in the periportal area, whereas fibulin-2 is expressed in myofibroblasts.

Although the cells responsible for the increased synthesis and deposition of ECM during fibrogenesis in the liver are not only HSCs but also endothelial cells and other mesenchymal cells ([52], reviewed in [53]), BM-derived cells migrating into fibrotic tissue of the liver have recently been noted [54,55,56]. BM-derived cells exhibit the features of collagen-producing cells such as HSC, myofibroblasts, and fibrocytes and seem to participate in the progression of liver fibrosis. These findings were observed by BM transplanted with sex-mismatched cells [54,55] and BM-derived marked cells with enhanced green fluorescent protein (EGFP) [56,57,58]. When α-SMA-positive myofibroblasts are observed, there is no clear evidence that these cells are producing collagen and contributing to the progression of liver fibrosis (reviewed in [59]). Thus the present author’s group sought to evaluate the direct contribution of BM-derived cells to collagen production by using the following specific and quantitative methods ([27], reviewed in [59]). Wild-type mice were irradiated and their BMs destroyed. The irradiated mice were then transplanted by BM from transgenic animals having tissue-specific enhancer/promoter sequences of α2(1) collagen gene (COL1A2) linked to EGFP or firefly luciferase (LUC) gene. We observed a large number of EGFP-positive collagen-expressing cells in liver tissue of transgenic COL1A2/EGFP mice in both liver fibrosis models, introduced by repeated CCl_4_ injections or ligation of the common bile duct. However, we observed few EGFP-positive BM-derived collagen-producing cells in the two liver fibrosis models of recipient mice. Luciferase assay also confirmed that BM-derived cells produced little collagen in response to fibrogenic stimuli [27]. Although BM-derived cells may differentiate into collagen-producing cells depending on the etiology and the extent and timing of tissue injury, we showed negligible contribution of BM-derived cells to collagen production during hepatic fibrogenesis in mice using very sensitive and specific methods [27].

Macrophages have recently been shown to play a role in the resolution [60] as well as in the fibrogenesis of experimental mouse fibrosis [61]. Hepatic macrophages (HM)-derived IL-1 and TNF-α activated NF-κ and prevented HSC death in *in vivo* and *in vitro* studies. IL-1 and TNF-α did not promote HSC activation but promoted survival of activated HSCs *in vitro* and *in vivo* and thus increased liver fibrosis. Xie *et al.* [62] showed that the signals of both Notch and Hedgehog pathways affect key cells participating in the tissue repair in adult liver and modulate epithelial-to-mesenchymal-like/mesenchymal-to-epithelial-like cell translations.

Chemical reagents, such as acetaldehyde, metabolized after alcohol consumption, have been considered to stimulate the *initiation* phase of HSCs activation in alcoholic liver diseases (ALD). Rapid transcriptional changes in HSCs induce cell response to cytokines and other stimuli, such as acetaldehyde. The next problem is “*reactive oxygen species (ROS)*” generated either from metabolically impaired hepatocytes or activated Kupffer cells seen in steatosis and steatohepatitis. In ALD, even though ROS can by itself activate HSC, cytokines or lipid oxidation products may also be involved in the activation. Abundance of proinflammatory cytokines, including TNF-α and IL-1 or IL-6, induces and perpetuates HSC activation followed by the release of neutrophils and monocyte chemoattractants and upregulation of adhesion molecules. Gut-derived endotoxin, endotoxin-recognizing receptors (CD14, TRL4, MD2), and endotoxin-induced activation pathways of NFκB and JNK have been observed in HSC activation in ALD. Augmented ethanol-induced ROS production in CYP2E1-overexpressing HSC leads to enhanced collagen α(1) gene expression [63]. ROS modulates the activity of transcription factors involved in HSC activation and fibrogenesis, e.g., c-Jun/AP-1, NF-κB, SP1 or c-Myb [63]. Lactic acid resulting from the cellular redox state in the liver by the ratio of NAD/NADH and NADP/NADPH, has been known to induce HSC activation and fibrogenesis. NADPH oxidase, mainly expressed in activated Kupffer cells, may activate HSC by generating H_2_O_2_, which induces collagen α(1) gene upregulation [64]. Hypoxia-inducible factor-1 is also seen in ALD and upregulates the transcription of VEGF. Acetaldehyde can directly upregulate collagen genes (reviewed in [46]).

### 4.2. Fibrogenesis in NASH

Progression of fibrosis was noted in 26% to 37% of NASH patients during a follow-up period of up to 5.6 years, with up to 9% progressing to cirrhosis (reviewed in [4]). The reason why the deposition of TG causes inflammation in the liver has been investigated [41]. Increased exposure of hepatocytes to saturated fatty acids can trigger inflammation by interacting with TLRs (described later) and apoptosis by activating death receptors [65] (Table 1). Saturated fats can also inhibit mitochondrial function and induce the ER stress pathway [65]. Diet-induced weight loss with increased physical activity has been shown to be associated with improvement of liver pathology [66]. It is known that NASH-related fibrogenesis develops from NAFLD due to multiple factors, such as insulin resistance, oxidative stress, pro-inflammatory cytokines and adipokines and innate immune responses. HSC is the main player in development of fibrogenesis in NASH and the activation mechanisms of HSC have been investigated in experimental studies ([67,68,69,70,71], reviewed in [72,73,74]) and human studies ([75,76,77,78], reviewed in [72,73,74,80]). Paradis *et al.* [67] revealed that high glucose and hyperinsulinemia stimulate connective tissue growth factor expression, and showed increased type I collagen expression in HSCs. In human studies insulin resistance is closely associated with the advanced stage of liver fibrosis in NASH patients, and the fibrosis is partially reversed by treatment with insulin sensitizers, such as pioglitazone, rosiglitazone and metformin ([69,75,76,77], reviewed in [72,73,74]).

**Table 1:** Reported factors involved in fibrogenesis of NASH.

Factors	Reference No.
Apoptosis of hepatocytes due to the deposition of TG	[41]
due to activated death receptors	[65]
Insulin resistance	[69,72,73,74,75,76,77,78]
Oxidative stress	[68,69,73,75,79]
Pro-inflammatory cytokines	[67,68,69,70,71,72,73,74,75,76,77,78,80,81,82,83]
Adipokines including leptin	[70,72,73,74,80]
Innate immune responses including TLRs	[73,74,84,85,86,87,88,89,90]
Connective tissue growth factor due to high glucose level	[67]
due to hyperinsulinemia	[67]
Liver fatty acid binding protein (L-Fabp)	[91]
Farnesoid X receptor (FXR)	[73,79]
Peroxisome proliferator-activated receptors (PPARγ)	[47,74,77,92]
MCP-1, CCR2	[93]
Bone-marrow-derived macrophages (Ly6C)	[93,94]
Hepatic stem/progenitor cells (HPCs)	[95,96,97,98,99]

Liver fatty acid binding protein (L-Fabp) has recently been noted. L-Fabp modulates HSC fatty acid utilization and regulates the fibrogenic genes. L-Fabp deletion inhibits HSC activation and attenuates both diet-induced hepatic steatosis and fibrogenesis [91]. L-Fabp appears to be useful in differentiating NASH patients from patients with simple steatosis.

Oxidative stress in NAFLD based on lipid peroxidation in mitochondria and peroxisomes induces activation of HSC. Ikeda *et al.* [68] showed the relation between ROS seen in a NASH model and HSC activation, increased mRNA expression of type I collagen and MMP-2 through the p38/MAPK signaling pathway. Li *et al.* [69] reported that ROS derived from hypoxic hepatocytes regulates MMP-2 expression in HSC. CYP2E1, as in ALD, generates oxidative stress in NAFLD, and activates HSC with the increased secretion of type I collagen, and this process was blocked by anti-oxidants and CYP2E1 inhibitors (reviewed in [73]). Sanyal *et al.* [75] showed effective results of vitamin E as an anti-oxidant in NASH patients, and McCarty [76] reported the useful effect of astaxanthin [76]. In patients with NAFLD daily fructose ingestion is associated with increased fibrosis (reviewed in [74,80]). Mice maintained on a high-fat and high-fructose diet in addition to developing obesity also showed increased hepatic ROS formation and a NASH-like phenotype with significant fibrosis (reviewed in [74,80]). Recently Inagaki’s group succeeded in demonstrating the direct contribution of mitochondrial oxidative stress to hepatic fibrogenesis using “Tet-mev-1 mouse” in which a mitochondrial reactive oxygen species can be induced by deoxycycline-regulable expression of mutant succinate dehydrogenase [79].

Mari *et al.* [81] found that loading of free cholesterol (FC) on mitochondria sensitizes cells to TNF- and Fas-induced steatohepatitis, but this mechanism was not observed with the loading of free fatty acids or triglycerides. Free cholesterol accumulated in hepatocytes exacerbated LPs-stimulated acute liver injury followed by apoptosis through TNF-α. Teratani *et al.* [82] pointed out that TNF-α mediated hepatocytes apoptosis was not involved in the progression of liver fibrosis. They observed that a high cholesterol diet aggravated a mouse liver fibrosis model induced by BDL or CCL_4_. Exacerbation of liver fibrosis was clearly caused by HSC accumulated in FC which sensitized HSC to TGFβ-induced activation.

As noted above, NAFLD is frequently found among patients with obesity, type 2 diabetes and metabolic syndrome. The adipocytes, inflammatory cells including macrophages and other monocytes secrete adipokines and pro-inflammatory cytokines. Adipokines include adiponectin, leptin, resistin, TNF-α, IL-6, visfatin, chemerin and vaspin. Adipokine receptors, AdipoR2 and AdipoR1, are present in the liver and skeletal muscle. AdipoR2 is known to play an important role in NAFLD because AdipoR2 expression decreased in a rodent NAFLD model fed a high-fat and cholesterol-rich diet followed by inflammation and fibrosis. Adiponectin has antifibrogenic effects in liver injury, and may act to reverse HSC activation and abrogates TGF-β1 signal transduction (reviewed in [72,73,74,80]). Adiponectin knockout mice showed more severe pericellular fibrosis compared with WT mice [70]. The advanced stage of NASH with fibrosis to cirrhosis sometimes showed reduction of hepatic fat (burn-out NASH) as described above. Liver fat loss often accompanies advanced fibrosis and cirrhosis. Van der Poorten *et al.* [9] clarified that the circulating adiponectin levels have an inverse correlation with hepatic fat content. As hepatic fat declines with advanced fibrosis, adiponectin levels progressively rise, independent of insulin resistance, leptin, BMI and waist/hip ratio. Adiponectin, in part, signals through phosphorylation of activated protein kinase and acetyl-CoA carboxylase to reduce lipogenesis. Increased levels of bile acids are seen in late-stage NASH, and bile acids act directly to regulate adiponectin synthesis in adipocytes [9]. As new anti-fibrogenic factors the liver X receptor (LXR) ligands, the farnesoid X receptor (FXR) and GW4064 (agonist of FXR) have recently been reported (reviewed in [73,80]). FXR is a bile acid sensor that functions to protect the liver and the intestine against bile acid toxicity and regulate synthesis, uptake and excretion of bile acids. As the bile acid pool size can affect lipid metabolism, FXR is considered to play a key role in lipid homeostasis by reducing both hepatic lipogenesis and plasma triglyceride and cholesterol levels (reviewed in [80]). The activity of peroxisome proliferator-activated receptors (PPARs) is involved in HSC activation, and PPARs play a key role in fibrogenesis of NASH (reviewed in [47,100]. PPARγ maintains the quiescent state of HSC phenotype (reviewed in [47]), and PPARγ agonists such as pioglitazone and rosiglitazone recovered to the quiescent phenotype from myofibroblast-like cells of HSC followed by decrease in NASH fibrosis in experimental and clinical studies ([74,77,92], reviewed in [47]), although Belfort *et al.* [101] showed no decrease in NASH fibrosis, but some decrease in inflammation in liver.

Miura *et al.* [93] showed that the Kupffer cell depletion ameliorated steatohepatitis with a decrease in MCP-1 and CCR2 expression, and that bone marrow-derived macrophage (Ly6C) decreased at the onset of an experimental NASH model. A differential contribution of Kupffer cells and blood monocytes during the development of NASH was shown as follows. TNFα producing-Kupffer cells appeared on day 2 after starting a methionine/choline-deficient diet, resulting in the infiltration of bone marrow-derived monocytes (CD11b^int^Ly6C^hi^) at day 10. Knockdown TNFα expression in bone marrow-derived cells ameliorated NASH development, *i.e.*, Kupffer cells producing TNF-α play an important role in the early phase of the development of NASH [94].

Leptin, an anti-obesity peptide hormone, is primarily secreted by adipocytes, but can also be produced by non-adipocyte cells, including HSCs, and leptin is a potential pro-fibrogenic adipocytokine (reviewed in [72,73,74,80]). Choi *et al.* [71] revealed that leptin promoted the phenotypic transition of HSCs by activating the Hh pathway followed by the development of liver fibrosis, that is, by the activation of the PI3K/AKT and JAK/STAT signaling pathways via binding to ObR (leptin receptor) followed by the activation of Hh pathways which also induce osteopontin leading to fibrosis progression in NASH [102]. Leptin is also involved in modulation of the angiogenic effect on activated HSC, and both leptin and PDGF increased the expression of HIF-1α and VEGF mediated by mammalian target of rapamycin (mTOR) via NADPH-oxidase in HSC [83]. Serum levels of leptin are elevated in NASH patients (reviewed in [73,80]), and Medici *et al.* [78] observed that serum levels of soluble leptin receptor were correlated with the stage of fibrosis in NAFLD patients. Visfatin expression in the liver is also reported to be high in NASH patients and correlated with the stage of fibrosis (reviewed in [73]).

Toll-like receptors (TLRs) can recognize molecular patterns of microbiological pathogens and signals of adaptor molecules such as myeloid differentiation factor 88 (MyD88) followed by the activation of NF-κB, AP-1, interferon regulatory factors (IRFs), *etc.*, and the implementation of the liver innate immunity (reviewed in [84]). The knockout of TLR4 inhibited lipid accumulation and mRNAs of ECM in the liver tissue of steatohepatitis mouse models. Obesity increases sensitivity to low doses of endotoxin lipopolysaccharide (LPS) derived from bacteria cell walls in the gut and leads to steatohepatitis ([85], reviewed in [84]). This finding indicates the interrelation between the LPS and the pathogenesis of NASH, and between LPS and the activation of HSCs through binding to TLR4, TLR9 and other TLRs followed by HSC proliferation and collagen production ([86], reviewed in [73,74,84]), as well as between endothelial TLR4 and fibrosis-associated angiogenesis [87] and between TLR4 and angiotensin-II in fibrogenesis of NASH [88]. Inflammasomes, potent inducers of IL-1β and IL-18 during inflammation, are large protein complexes consisting of a Nod-like receptor (NLR) and PYHIN proteins, and function as sensors of endogenous pathogen-associated molecular patterns (PAMPs) that regulate the release of inflammatory cytokines such as pro-IL-1β and IL-18. Most PAMPs activate NLRP3 inflammasome. Henao-Mejia *et al.* [89] reported that inflammasome deficiency causes a pattern change of gut micropathogens followed by increased influx of TLR4 and TLR9 agonists into the portal circulation and exacerbation of hepatic inflammation and steatosis with TNF-α expression, leading to NASH progression. The activation of TLR4 signaling in hepatocytes accompanied by the relocation of P65 in nucleus was proven to be involved in the initiation of NAFLD. High-mobility group Box1 (HMGB1) released from hepatocytes in response to free fatty acid infusion was found to be the key molecule for the TLR4/Myd88 activation and cytokines expression *in vivo* and *in vitro* [90].

Hepatic stem/progenitor cells (HPCs) are involved in the adaptive response to injured hepatocytes by oxidative stress in NAFLD (reviewed in [95]). Activation of HPCs causes ductular reaction via activation of the Wnt pathway followed by HPC expansion in the periportal area and portal fibrosis in the repair process of liver damage. Van Hul *et al.* [96] showed HPC expansion and ECM accumulation in a choline-deficient, ethionine-supplemented (CDE) model of HPC proliferation. HPCs proliferate in the periportal area, migrate inside the lobule and undergo further differentiation in chronic liver injury. In this CDE model, collagen deposition was observed after day 3, and increased numbers of cytokeratin 19 (CK19)-positive cells at day 7. At day 3 matrix-producing cells occurred as an initial phase prior to HPC expansion. Moreover, cellular cross-talk and molecular network of HPCs, hepatocytes, HSCs, and macrophages develop by Notch and Wnt signaling, which direct HPC specification within the activated myofibroblast and macrophage HPC niche leading to the formation of NASH and fibrosis ([97], reviewed in [95]). HPC activation is correlated with fibrosis and the progression toward NASH, as revealed in pediatric patients [98]. A review by Friedman [99] clearly showed that gut microbiome stimulation caused hepatic injury and fibrosis through specialized signaling complexes combined with increased Hh activity and HPC expansion in NASH.

## 5. Role of MMPs and TIMPs in Fibrogenesis of NASH

MMPs and TIMPs in fibrogenesis of NASH may play a role in contributing not only to the balance between the formation and the degradation of connective tissue components, but also to the signal transduction for tissue recovery to normal condition. In the largest longitudinal study of paired liver biopsy samples, a stable clinical course was noted in 34% to 59% of patients with NASH, and improved histology was noted in 16% to 29% ([39], reviewed in [8]). MMPs and TIMPs are critical in the clinical course of NASH.

### 5.1. MMPs and TIMPs in Progression from Liver Fibrosis to Cirrhosis

In 1974 Okazaki and Maruyama [14] demonstrated collagenolysis around the explant of a slice of rat fibrotic liver on a collagen gel film, and showed the typical collagenase attack pattern. Subsequently, collagenase activity for type I collagen in homogenate samples of baboon and human livers, under neutral pH and addition of 3 mM p-chloromercuribenzoate to inhibit thiol proteinase activity and to convert procollagenase into the active form, was measured by viscometer at 27 °C monitored by disc electrophoresis, which clearly showed β^A^, α^A^ and α^B^ [15,16]. The levels of the reaction products of β^A^ and α^A^ increased in the early stage of hepatic fibrosis in baboons subjected to ethanol feeding over several years and in patients with alcoholic fatty liver [17]. Subsequently other researchers in biochemical and histological studies reported increased collagenase activity in the early stage of liver fibrosis and reduced collagenase activity in advanced fibrosis (reviewed in [103]).

Arthur *et al.* [104] reported that HSCs secrete a neutral metalloproteinase that can degrade type IV collagen. The enzyme they observed seems to be MMP-2 and MT1-MMP-2. Takahara *et al.* [105] showed that the level of MMP-2 expression increased during the process of experimental hepatic fibrosis as well as during the process of hepatic fibrosis in chronic hepatitis, and that it decreased during the process of cirrhosis. Takahara *et al.* [106] also demonstrated the dual expression of MMP-2 and MT1-MMP in chronic hepatitis and cirrhosis, and further demonstrated cytoplasmic and membranous immunodeposits of both MMPs in endothelial cells, Kupffer cells, capillary endothelial cells and lymphocytes. In particular they observed the over-expression of MMPs in HSCs and fibroblasts and suggested that MT1-MMP activates pro-MMP-2. These MMPs may remodel the liver parenchyma during the process of liver fibrosis. MMP-2 and MT1-MMP have been considered to be fibrogenic enzymes because MMP-2 expression is stimulated by TGF-β while MMP-1 expression is down-regulated by TGF-β (reviewed in [107]).

Iredale *et al.* [108] did not observe increase in MMP-13 mRNA transcription in the experimental liver fibrosis of rats. Instead, they demonstrated an increase in TIMPs mRNA transcripts and postulated that the balance between the down-regulation of MMP-13 expression and the up-regulation of the expression of TIMPs may result in the deposition of type I collagen in experimental hepatic fibrosis. Discontinuation of chronic CCl_4_ administration causes decrease in TIMPs and relative increase in MMP-13 followed by degradation of fibrosis. At the same time apoptosis of HSC, that is, attenuation of ECM production by HSC, was seen. Yoshiji *et al.* [109] found that enforced expression of TIMP-1 using transgenic mouse diminished apoptosis of HSC and decreased MMP-2 activity followed by the remaining fibrosis after the discontinuation of toxic agents.

MMP-1 expression had not been observed in the liver. The present authors’ group attempted to observe gene expression of MMP-1 in the process of hepatic fibrosis in rats treated with CCl_4_ for 12 weeks as well as in the recovery phase, 2, 5 and 7 days after the last injection. The deposited ECM decreased dramatically after cessation of chronic CCl_4_ intoxication (reviewed in [29,103]). We found that gene expression of MMP-13 (the major interstitial collagenase in rodents) appeared clearly but transiently in the early stage of the recovery phase using Northern blotting and *in situ* hybridization [20]. In rats treated with CCl_4_ for 8 weeks, signals for MMP-13 mRNA were observed in a few cells at the interface between the resolving fibrous septa and the parenchyma. Some of these cells were stained with α-SMA, a marker of activated HSCs, in serial specimens of the liver in the very early recovery stage. It has been suggested that HSCs may play an important role in the degradation of ECM in the liver. On the other hand, the cirrhotic liver of rats treated with CCl_4_ for 12 weeks revealed very weak expression of MMP-13 mRNA in HSCs. No hepatocytes in the liver revealed MMP-13 mRNA transcripts regardless of the length of CCl_4_ treatment ([20], reviewed in [29,103]). Most cells positive for MMP-13 mRNA, however, were negative for markers of HSC, Kupffer cells and endothelial cells [20]. We then speculated about the possible contribution of bone marrow (BM)-derived cells to MMP-13 expression [24].

We hypothesized that these cells may originate from stem cells derived from bone marrow and prepared mice transplanted with enhanced green fluorescent protein (EGFP)-expressing BM cells after radiation. We then injected CCl_4_ repeatedly and observe spontaneous resolution of liver fibrosis [24]. A large number of EGFP^+^ cells showing BM-derived stem cells were seen in the fibrotic liver at the time of peak fibrosis, then gradually decreased with the resolution of fibrosis. MMP-13-expressing cells were observed mainly in the portal areas, and approximately half of them co-expressed EGFP, indicating BM origin. We checked the cell markers for both EGFP- and MMP-13-positive cells, but they were negative markers for HSC, Kupffer cells and endothelial cells. This study showed the fate, distribution and phenotype change of BM cells, and BM cells co-expressed with MMP-13 were observed at day 2, followed by BM cells co-expressed with MMP-9 observed at day 5 in the recovery phase. Overall, approximately half the MMP-13 positive cells co-expressed EGFP. In the original paper [24], using three colored markers, the MMP-9 (red)-expressed BM (EGFP; green)-derived cells differentiated into three lineages: (1) granulocytes, (2) F4/80 (blue), suggesting macrophage/Kupffer cells, and (3) CD34 (blue), probably hematopoietic or hepatic progenitor cells. The three colored markers were clearly observed. Enzymatic activity of MMPs was demonstrated at day 5 via *in situ* zymography, and the activity was inhibited definitively by the addition of MMP inhibitor [24]. Moreover, administration of granulocyte colony-stimulating factor (GCSF) showed increased migration of BM-derived cells into fibrotic liver and improved resolution of liver fibrosis. Treatment of GCSF to transgenic mice of hepatocyte growth factor (HGF) expressed increased MMP-9 positive cells with decreasing fibrous septa [24].

### 5.2. MMPs and TIMPs in NASH

There are very few reports on MMPs and TIMPs in NASH including NASH-related cirrhosis and HCC. Ljumovic *et al.* [110] showed that MMP-9 and MMP-10 expression in NASH patients was higher in comparison to viral hepatitis C by measuring mRNA levels with semiquatative RT-PCR, while MMP-2 mRNA expression increased in patients with chronic HBV and HCV hepatitis. They showed different expression patterns of MMP-2, -9, -10, -11 between viral and non-viral chronic liver diseases. Moreover, D’Amico, *et al.* [111] showed significantly higher plasma levels of MMP-9 in NASH patients (69.0 ± 14.5 ng/mL (SD)) than in HCV-infected liver disease patients (61.7 ± 11.0) (healthy controls 39.7 ± 4.6), and different MMP-9 immunolocalization patterns in the two diseases; positive staining was seen on granulocytes and faint cytoplasmic immunolabelling on hepatocytes in NASH while positive staining was noted on the biliary canaliculi as well as epithelium of bile ducts and cytoplasm of hepatocytes in HCV-infected liver.

Leptin caused the down-regulation of MMP-1 mRNA in LX-2 cell as demonstrated by the synergistic pathway networks of JAK/STAT and JAK-mediated ERK1/2 and p38 [112]. However, Wanninger *et al.* reported that serum MMP-1 levels were more highly correlated with the degree of fibrosis in patients with NAFLD [113]. These observations suggest that the liver tissue of NASH has greater increased activity of MMPs compared with the activity of TIMPs, and the clinical course seems to be generally benign.

Concerning MMP-9, Tarrats *et al.* [114] found that TNF receptor increased MMP-9, and Wanninger *et al.* [113] showed that the increased activity of MMP-9 was not induced by hepatic steatosis but by hepatic inflammation and fibrosis using a rodent model of non-alcoholic steatohepatitis. This induction seemed to be related to the anti-inflammatory activity of adiponectin rather than its effect on hepatocellular MMP-9 expression.

## 6. Carcinogenesis in NASH

It is known that cirrhosis is linked to the development of HCC regardless of the underlying etiology of liver disease. However, all cases of NASH-related HCC do not show cirrhosis, as noted in Section 1 [11].

Ten percent of patients with NASH-related cirrhosis developed HCC after a median follow-up of seven years [115]. 25/195 (12.8%) of NASH-cirrhotic and 64/315 (20.3%) of HCV-cirrhotic patients developed HCC during a median follow-up of 3.2 years in the USA [116]. Yearly cumulative incidence of HCC has been shown to be 2.6% in patients with NASH-cirrhosis, compared with 4.0% in patients with HCV cirrhosis [116]. A review by Starley *et al.* [4] revealed a lower occurrence of HCC in NASH-cirrhosis than had been reported previously. From 4% to 27% of NASH progressed to HCC after the development of cirrhosis retrospectively. The occurrence of HCC among NAFLD is reported to be 0%–0.5% in longitudinal studies and the prevalence of HCC in NASH to be 0%–2.8% over time periods of up to 19.5 years (reviewed in [4]).

### 6.1. Carcinogenesis from Chronic Liver Diseases

Advanced HCC shows a large tumor mass composed of several small nodules (called “nodules in nodule”) surrounded by thick fibrous bands. HCC cells show different differentiation stages from one nodule to another, but exhibit the same differentiation stages in each single nodule. The size of early HCC is smaller than 2 cm in diameter and composed of well-differentiated and moderately differentiated cancer cells. Although portal tracts and fibrous septa are seen within the nodule of early HCC, these structures gradually disappear as the nodule grows in size. It has been observed that well-differentiated cells invade portal tracts and fibrotic bands and destroy these structures. This is known as “stromal invasion” (reviewed in [35]).

A multi-step hepatocarcinogenesis and subsequent progression have been clarified. The formation of atypical lesions increases in order from low-grade dysplastic nodule to high-grade dysplastic nodule, then to early HCC composed of well-differentiated cancer cells. Less differentiated malignant cells arise in the well-differentiated tumor and occupy and replace the well-differentiated tumor as the nodules grow in size. Moderately differentiated cancer cells appear within the group of well-differentiated cancer cells, compress them outward, and a fibrotic capsule between well- and moderately differentiated cancer cells is formed, resulting in a fibrous capsule surrounding advanced HCC. Advanced HCC with nodules in nodule with thick fibrous bands is formed in this manner (reviewed in [35]).

As previously noted, in the developed countries HCV-related HCC and HBV-related HCC decrease but NASH-related HCC increases. Even if the HCC has a viral etiology, insulin resistance, oxidative stress, inflammatory cytokines and autophagy contribute to the carcinogenic potential for chronic liver diseases (reviewed in [79]). Epithelial-mesenchymal transition (EMT) is associated with overexpression of p28^GANK^ [117] and Fastin-1 [118] followed by invasiveness and angiogenesis. Up-regulation of an oncoprotein, p28^GANK^, causes cell cycle progression in hepatocytes. This oncoprotein activates PI3K/Akt/HIF1α to increase expression of TWIST1, VEGF and MMP-2 [119]. Fascin-1 is an actin bundling protein and is considered to be a migration factor associated with EMT in HCC. When combined with MMPs it is thought to ease invasiveness [118]. Besides well-known cytokines such as VEGF, ILs and others, the inhibitory mechanism of adiponectin has been reported. Adiponectin inhibited not only MMP-9 expression but also the ROCK/IP10/VEGF signaling pathway, resulting in suppression of tumor angiogenesis and cell migration [120]. Human HSCs in culture release stromal derived factor-1 (SDF-1), and co-cultured cholangiocarcinoma (CCA) cells are induced by SDF-1 binding to CXCR4 followed by increased migration of CCA cells [121]. HSC is involved in primary and secondary carcinogenesis in the liver.

### 6.2. Carcinogenesis from NASH

NASH is a common underlying liver disease in patients with HCC [122], and obesity-related cryptogenic cirrhosis should be considered as a complication of HCC [123]. HCC is usually diagnosed in a late stage, but it may also occur in non-cirrhotic NASH [11]. HCC in patients with features of metabolic syndrome as the only risk factor for liver disease often showed absence of significant fibrosis in the background liver [124]. Ascha *et al.* [116] reported that age and daily alcohol consumption, even if the quantity was not so high within diagnosis criteria, were independent variables besides obesity and the complication of type 2 diabetes mellitus as risk factors of HCC in patients with NASH as revealed by multivariate regression analysis (Table 2). Many studies have reported that both obesity and diabetes independently increase the risk of developing HCC (reviewed in [3,4,5]). Chronic HCV patients with steatosis, obesity and type 2 diabetes mellitus show higher risk for HCC (reviewed in [4,5]). Iron deposition and advanced fibrosis have also been found to be risks for NASH-related HCC (reviewed in [4]). Fibrosis in NASH patients showed higher risk for HCC than patients with a lower degree of fibrosis (88% *vs.* 31%) [8].

**Table 2:** Risk Factors involved in Carcinogenesis of NASH.

Risk Factor	Reference No.
Age	[116]
Obesity	[3,4,5,79]
Type 2 diabetes mellitus	[3,4,5]
Fibrosis	[8,121,125,126]
Daily alcohol consumption	[116]
Lipid-modifying enzymes to produce MUFA*	[127]
Insulin resistance	[4]
Hypoadiponectinemia	[4,79]
Hyperinsulinemia	[79,128]
Oxidative stress, release of ROS	[4]
Inhibition of NF-κB	[4]
Absence of JNK1	[4]
TLRs	[91]

*MUFA: monounsaturated fatty acids.

Regarding abnormal lipid metabolism related with HCC in NASH, the following experimental result was recently reported by Muir *et al.* [127]. Pten-knockout mice develop NASH and subsequently NASH-derived HCC. In this model lipid-modifying enzymes converting saturated fatty acid (SFA) to monounsaturated fatty acids (MUFA) were demonstrated to play an important role in HCC because the ratio of long chain n6-polyunsaturated fatty acids over n3-polyunsaturated fatty acids showed higher risk of HCC among NASH mice.

Generally the following factors were listed in tumorigenesis from NASH. Obesity and insulin resistance result in decreased amounts of adiponectin and increased proinflammatory cytokines such as TNF-α and IL-6 (reviewed in [4,80]). Adiponectin has been shown to be an anti-inflammatory polypeptide secreted from adipose tissue that improves insulin resistance. It has been shown that hypoadiponectinemia accelerated hepatic tumor formation and inhibited angiogenesis via modulation of apoptosis in a NASH mouse model (reviewed in [4,80]). Hyperinsulinemia upregulates the production of insulin-like growth factor-1 and hepatocyte growth factor (HGF), leading to HCC progression. Insulin activates the insulin receptor substrate-1 (IRS-1), and IRS-1-mediated signals may act as survival factors and protect against TGF-β-induced apoptosis in HCC cell lines. Overexpression of IRS-1 activates MAPK and PI3K followed by hepatocyte proliferation and growth of HCC (reviewed in [80,128]). Moreover, the mannose 6-phosphate/IGF2 receptor (M6P/IGF2R) has been demonstrated to function as a tumor suppressor because this receptor inhibits cell growth factors and inactivates IGF2, a growth stimulator. In fact, mutations of this receptor have been revealed in 61% of HCC patients (reviewed in [4]).

Oxidative stress and the release of ROS contribute to the development of both NASH and HCC. The oxidative stress may cause HCC via inflammation and cell proliferation or via inducing cancer promoting gene mutation. In an obese mice model proven to be insulin-resistant, mitochondria in hepatocytes with steatosis showed increased production of ROS, and oxidative stress seems to be involved in hepatocyte hyperplasia. Epithelial hyperplasia and/or morphological dysplasia change lead to the development of cancer after a long period of time (reviewed in [4]). Patients with high levels of 8-OHdG and 4-HNE revealed a higher incidence of recurrent HCC (reviewed in [4,80]). Oxidative stress induces gene alterations to cause cancer directly. For example, *trans*-4-hydroxy-2-nonenal, a metabolite of lipid peroxidation, has been demonstrated to cause mutation of the p53 suppressor gene (reviewed in [4]). Hepatocytes lacking nuclear respiratory factor-1 (Nrf1), an essential transcription factor mediating oxidative stress, increase susceptibility to oxidative stress. Cases lacking Nrf1 showed steatosis, apoptosis, necrosis, inflammation and fibrosis, and developed hepatic cancer. All these alterations involve oxidative stress (reviewed in [4]).

NF-κB regulates immune and inflammatory responses and is activated in many tumors, inhibiting apoptosis. Inhibition of NF-κB in mouse was shown to cause spontaneous apoptosis of hepatocytes followed by steatohepatitis and subsequent HCC (reviewed in [4]). JNK1 activation increases hepatic inflammation and apoptosis. Patients with NASH have significantly increased phosphorylated JNK1 levels compared to patients with benign NAFLD. The absence of JNK1 prevented weight gain and development of insulin resistance. JNK1 is overactivated in more than 50% of human HCC samples. The overactivation of JNK1 leads to increase in several genes for hepatocyte proliferation and risk of HCC (reviewed in [4]).

Evidence is accumulating regarding a close relationship between fibrosis and HCC, although this association remains unclear. The major evidence includes the involvement of inflammatory cells, integrin signaling, growth factor interactions with the ECM, and communication between activated HSC and cancer cells (reviewed in [125,126]). Although HSC is involved in primary and secondary carcinogenesis in the liver as described above [121], Yin *et al.* [126] note that HSC may be involved in HCC development in the dysregulation of liver regeneration process, *i.e.*, in the early phase of liver regeneration the activated HSCs promote the proliferation of HPCs and hepatocytes, stimulate angiogenesis via the activation of endothelial cells including transdifferentiation to endothelial cells from HSCs, and in the late phase HSCs may undergo a mesenchymal-to-epithelial transition to HPCs, then to hepatocytes or biliary cells, and finally to cancer cells.

Extracellular matrix remodeling and epithelial cell-to-mesenchymal transformation (EMT) are considered to be involved in hepatocarcinogenesis and metastasis. Hh signaling promotes EMT during adult liver repair and regeneration, including ductular progenitors and HSC to convert into myofibroblasts. Hh signaling contributes to carcinogenesis via growth of the progenitor cell population (reviewed in [129]). The following recent reports are not of direct research on NASH-related tumorigenesis but contain interesting findings. Weiskopf *et al.* [130] identified FD6 and CV1, which block the “don’t eat me” signal secreted from macrophages. Yoshimoto *et al.* [131] showed that senescence-associated secretory phenotype (SASP) played an important role in promoting obesity-associated HCC development in mice. Deoxycholic acid stimulates SASP phenotype in HSCs, causing the secretion of various inflammatory and tumor-promoting factors in the liver, leading to HCC. TLRs exhibit different roles in the regulation of tumorigenesis [91]. Lin *et al.* [132] found that the genetic deletion of TLR2 increased susceptibility to dimethylnitrosamine to cause HCC. TLR2 deficiency caused decrease in the infiltration of macrophages and attenuation of apoptosis and ASK1/p38 MAPK/NF-κB signaling, which led to a decrease in the expression of IFN-γ, TNF-α, IL-1α/β, and IL-6 in combination with Cxcl-2, and suppressed the autophagy immune system with increased oxidative stress and p62 in liver. On the other hand, Wang *et al.* [133] reported that TLR4 increases the expression of Ku70, DNA repair protein, and acts to protect against HCC carcinogenesis. NASH-related hepatocarcinogenesis should be further investigated from the viewpoint of innate immunity. Another recent finding of interest is that the DNA methylation profile can distinguish patients with NAFLD from those with NASH and/or NASH-related HCC [134].

## 7. Role of MMPs and TIMPs in Carcinogenesis of NASH

Altered proteolysis in cancer leads to unregulated tumor growth, tissue remodeling, inflammation, angiogenesis, tissue invasion and metastasis. MMPs and TIMPs are involved in these events via signal pathways seen in tumorigenesis (reviewed in [35,107]).

### 7.1. MMPs and TIMPs in HCC

Transcripts for MMP-9 were detected in tumorous tissues in 16 of 23 HCC samples from surgical specimens, and 15 of the 16 positive samples showed stronger expression in tumorous tissues than in non-tumorous tissues [135]. The correlation of MMP-9 expression and the presence of capsular invasion by conventional microscopical observation suggested that MMP-9 closely participated in capsular infiltration in HCC. Histological demonstration for MMP-9 protein showed strong staining in HCC cells, especially in the marginal area of the tumorous tissue, stromal fibroblasts, epithelial cells of the bile ducts and vascular endothelial cells [135]. MMP-9 mRNA was expressed in HCC cells of 22 of 27 HCC samples by *in situ* hybridization [136]. Strong expression of MMP-9 mRNA was shown in HCC cells at the invasion sites of both capsules and portal veins. MMP-9 production (or mRNA expression) was seen in HCC cells [137,138] and might be involved in stromal invasion and metastasis within nodules. Some scattered stromal fibroblasts and endothelial cells expressed MMP-9 mRNA at weak levels [136,139]. In a mouse model of liver tumor, MMP-9, -10, and -12, MDM2 and p53 expression were induced after exposure to dimethylnitrosamine, which causes oxidative stress and genomic injury followed by hepatocyte apoptosis, necrotic cell death, early stage of carcinogenesis, malignant cell invasion and migration [140].

Although MMP-2 involves angiogenesis in HCC [141], since Sato *et al.* [142] identified MT1-MMP and the activation mechanism of pro-MMP-2 by MT1-MMP and TIMP-2 on the cell surface was reported [143], there have been several papers investigating MMP-2, TIMP-2 and/or MT1-MMP in HCC [135,144,145,146,147,148,149,150,151,152,153,154]. Most [137,139,146,150,151,152,153,154] showed strong expression of mRNAs and proteins of both MT1-MMP and MMP-2 in both HCC cells and stromal cells in the invading border of tumor nests. On the other hand, Musso *et al.* [145] did not observe positive staining in HCC cells, but observed cells positive for MMP-2 mRNA(+)/TIMP-2 mRNA(+)/αSMA (+), which appeared to be stellate cells at the invasion front. This discrepancy was resolved by Ogata *et al.* [137], who observed MT1-MMP and MMP-2 in HCC and stromal cells. Both enzymes were detected within the same HCC cells. Moreover, these enzymes were associated with tumor dedifferentiation, *i.e.*, both enzymes were clearly detected in all poorly differentiated HCC, in 73% of moderately differentiated HCC, but MMP-2 was not detected in early (well-differentiated) HCC. MT1-MMP and MMP-2 mRNA were strongly expressed in the cytoplasm of HCC cells as well as in stromal cells surrounded by ECM in moderately and poorly differentiated HCC ([137], reviewed in [35]).

HCC cells were the main producers of MT1-MMP mRNA. Only a low level of expression was detected in stromal cells. The expression level in HCC cells varies from negative to strongly positive, showing an association with poorly differentiated HCC [139]. On the other hand, the expression of MMP-2 mRNA seen mainly in stromal fibroblasts and endothelial cells was poor in HCC cells. MMP-2 was abundantly synthesized by stromal cells, but it immunolocalized mainly to HCC cells [139]. The activation of MMP-2 is related to the coordinated high expression of TIMP-2, and MT1-MMP, but hepatocytes may also modulate the activation of MMP-2 through the expression of MT2-MMP [147]. MMP-12 (human macrophage metalloproteinase) not only degrades elastin and a broad range of matrix/non-matrix substrates, but also participates in generating angiostatin, an internal fragment of plasminogen with an angiogenesis-inhibiting function. *In situ* hybridization revealed MMP-12 mRNA in 25 of 40 HCC samples. Patients without positive findings did not produce angiostatin and demonstrated poorer survival than those with positive findings [155]. There was no relationship between the grades of HCC dedifferentiation and positive staining [155]. Advanced HCC cases showed no MMP-1 mRNA expression ([32], reviewed in [35]).

Well-differentiated cancer cells in early HCC are known to invade portal tracts and/or fibrous bands resulting in the disappearance of these fibrous tissues ([32], reviewed in [35]). Sakamoto *et al.* [138] used semi-quantative RT-PCR to investigate the mRNA expression of both MMP-2 and MMP-9 in 37 pairs of HCC and adjunct non-tumor tissue specimens, and confirmed that MMP-9 overexpression was correlated with growth of small HCC. Ogata *et al.* [137] used samples of well-differentiated HCC smaller than 10 mm in diameter obtained by ultrasound-guided fine-needle biopsy, and detected MT1-MMP in one of the six well-differentiated HCCs, but MMP-2 was not found in any of these same samples.

The present authors hypothesized that the degradation of ECM by MMP-1 might be involved in the process of cancer cell invasion. We investigated the localization of both mRNA and protein of MMP-1 by *in situ* hybridization and immunohistochemical staining, respectively, in seven cases of early HCC smaller than 2 cm in diameter and compared them with those of seven cases of advanced HCC [32]. Four of seven cases with early HCC showed only well-differentiated cancer cells, two cases showed both well-differentiated and moderately differentiated cancer cells, and the remaining case showed only moderately differentiated cancer cells. *In situ* hybridization revealed that three of four cases with only well-differentiated HCC expressed MMP-1, two cases with both well-differentiated and moderately differentiated HCC showed positive staining, and one case with only moderately differentiated cells showed negative staining. No case of advanced HCC showed MMP-1 mRNA. The positive cells were well-differentiated cancer cells located at the invading front of the cancer. An interesting finding was that positive cells were scattered in a ratio of approximately 1% to 2% in cancer. This was very different from the findings of MMP-9 expression reported in other studies described above. Hepatocytes of non-cancerous liver did not express transcripts of MMP-1. Positive staining of MMP-1 protein was seen in early HCC with well-differentiated HCC cells and invaded portal tract. In another case with early HCC, well-differentiated HCC cells positive for MMP-1 protein were compressed by moderately differentiated cancer cells which were negative for MMP-1 staining. MMP-1 mRNA was seen in HCC cells infiltrated into the small portal tract left in tumor nodule as well as in the fibrous bands surrounding the cancer cell nodule. MMP-1 expression was associated with growth of small HCC in which well-differentiated cancer cells invade the portal tract and fibrous bands, and these fibrous tissues disappear upon participation of MMP-1 [32].

Nakatsukasa *et al.* [156] reported that TIMP-1 mRNA and TIMP-2 mRNA in cancerous tissue were homogeneously stained more intensively than in non-tumorous tissue of HCC by *in situ* hybridization. All HCC tissues contained transcripts of both TIMP-1 and TIMP-2 with stronger expression in HCC cells than in the surrounding tissue. The expression and distribution of the transcripts for TIMP-1 and TIMP-2 did not differ among the various cancer differentiation stages. The intensity of TIMPs mRNA expression varied from nodule to nodule. Stromal cells in and surrounding the HCC expressed both TIMP-1 and TIMP-2 mRNA, and their expression in stromal cells present in the capsule was especially strong. On the other hand, Musso *et al.* [145] did not observe either mRNA of MMP-2 or TIMP-2 in HCC cells, but observed them in αSMA-positive cells at the invasive front. The MMP-2(+)/TIMP-2(+)/αSMA(+) cells in the perisinusoidal space adjacent to liver tumors are considered to be HSC. This discrepancy is probably due to the histological differences in HCC used in the study above. TIMPs act to modulate the matrix/tumor interaction [138,139,140,142]. Furthermore, TIMPs may play an important role in cell growth, and pro-MMP-2 may be activated by MT1-MMP and TIMP-2 on the cell surface of HCC cells, stromal cells and stellate cells resulting in stromal invasion. TIMP-1 and TIMP-2 expression by stromal cells was associated with a poorer prognosis of HCC as revealed by an immunohistochemical study using tissue microarrays [153,154].

**Figure 2:**
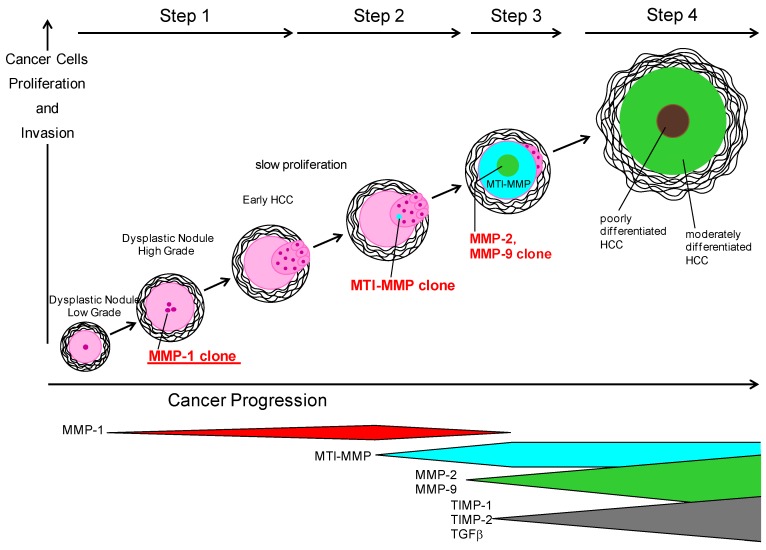
MMPs and TIMPs expression related with HCC progression based on the reported results in HCV-derived HCC [35]. The terminology of nodular hepatocellular lesions in this figure is modified by the report of the International Working Party [157]. Step 1: A conversion from low-grade dysplastic nodule to high-grade dysplastic nodule within liver cirrhosis obtains a phenotype to express MMP-1, resulting in the formation of a new clone. Early HCC cells proliferate slowly with the ability of stromal invasion. Step 2: New clones can proliferate and invade portal tracts and fibrous tissue. Subsequently other clones arise to degrade effectively not only fibrous tissue but also basement membrane. These clones of the next generation express MT1-MMP in the process of cancer development. Step 3: Several early HCC cells express MT1-MMP and gradually small amounts of MMP-2 and MMP-9, probably stimulated by inflammatory cytokines or TGF-β, which may participate in the stromal invasion or the formation of the thick capsule of cancer nodules. Pro-MMP-9 is activated by MMP-2, or early HCC to moderately differentiated HCC cells obtain a phenotype expressing MMP-9. TIMP-1 and TIMP-2 gene transcripts in HCC cells increased with increased expression of MMP-2 and/or MMP-9. Step 4: MMP-1 positive clones (early HCC cells) are compressed by new clones (moderately differentiated hepatoma cells) and subsequently disappear [35].

The above observations on MMP-1, -2, -9, MT-1 MMP and TIMP-2 lead to the following hypothesis, as shown in Figure 2 [35]. The conversion from low-grade dysplastic nodule to high-grade dysplastic nodule within liver cirrhosis results in a phenotype that expresses MMP-1, resulting in the formation of a new clone. Well-differentiated HCC cells proliferate slowly with the ability of stromal invasion ([32], reviewed in [35]). New clones can proliferate and invade portal tracts destroying fibrous tissue. Subsequently other clones arise to degrade effectively not only fibrous tissue but also basement membrane. These next generation clones express MT1-MMP in the process of cancer development. Several well-differentiated HCC cells express MT1-MMP and gradually small amounts of MMP-2 and MMP-9, probably stimulated by inflammatory cytokines or TGF-β, which may participate in the stromal invasion or the formation of the thick capsule of cancer nodules. Pro-MMP-9 is activated by MMP-2, or well- to moderately differentiated HCC cells to obtain a phenotype expressing MMP-9. TIMP-1 and TIMP-2 gene transcripts in HCC cells appeared with increased expression of MMP-2 and/or MMP-9. MMP-1-positive clones (well-differentiated cancer cells) are compressed by new clones (moderately differentiated cancer cells) and subsequently disappear.

There is no direct evidence for this hypothesis. However, we have previously reported that stem cells derived from bone marrow changed their phenotypes of MMP-13 (a major interstitial collagenase in rodents with homology to human MMP-1) to MMP-9 expression in the recovery phase from experimental liver fibrosis and cirrhosis [24]. A similar switch in MMP expression may occur in the dedifferentiation process of cancer stem cells (Figure 2). On the other hand, positive staining for both MMP-2 and MMP-9 proteins was observed in inflammatory cells, fibroblasts and endothelial cells. MMP-9 mRNA was detected in mesenchymal cells inside and outside cancer nodules, and in fibrous capsules around the necrosis of cancer nodules [128]. Mesenchymal cells and inflammatory cells positive for MMP-2 and MMP-9 were numerous and stained strongly for both mRNA and protein compared with cells positive for MMP-1 [32,145,147]. These positive cells may participate in the degradation of the fibrous tissue to allow HCC cells to invade easily or to form the thick capsule around the nodule.

Cancer-associated mesenchymal stem cells (MSC) could cross-talk with tumor cells and secreted many growth factors and chemokines, which play crucial roles in tumor progression [158,159]. A recent report by Yan *et al.* [160] clearly demonstrated the presence of liver cancer-associated MSCs (LC-MSC) which promote the proliferation and metastasis of HCC cells. They indicated that S100A4 secreted from LC-MSCs can promote HCC cell proliferation and invasion, and the invasion-promoting effect of S100A4 was attenuated by a miR-155 inhibitor. MiR-155 induced by S100A4 increased MMP-9 expression; S100A4 facilitated the process by up-regulating MMP-9. S100A4 and miR-155 expression and down-regulating SOCS1 gene expression, which regulates p-STAT3, one of the mediators involved in regulating MMP-9 expression, stimulate HCC invasion. Moreover, Roderfeld *et al.*, demonstrared that MMP-9 expressing macrophages invade at the tumor front of HCC [161].

Expression of MMPs is controlled mainly at the transcriptional level. The promoter regions of MMPs contain several common elements where the common transcription factors such as AP-1, Ets, and/or NF-κB bind (reviewed in [35]). The present authors showed that the c-Jun NH2-terminal kinase (JNK) pathway is involved in constitutive MMP-1 expression in a well-differentiated cell line (HLE cells) among five HCC cell lines derived from various dedifferentiation stages. C-Jun is phosphorylated by JNK, one of the four distinctly regulated MAPK pathways; the other three pathways are extracellular signal-related kinases (ERK)-1/2, p38 proteins and ERK5. HLE cells constitutively expressed MMP-1 gene and protein as well as its enzymatic activity without the use of any stimulators such as phorbol ester. MMP-1 gene transcription was controlled by the activation of c-Jun through the JNK pathway [33].

PTEN inhibits the migration and invasion of HCC by the down-regulation of MMP-2 and MMP-9 in a PI3K/Akt/MMP-dependent manner [162]. Lysophosphatidic acid (LPA) produced extracellularly by autotaxin (ATX), increases cell survival, angiogenesis, invasion and metastasis [35]. Park *et al.* [163] revealed that silencing or pharmacological inhibition of LPA1 inhibited LPA-induced MMP-9 expression and HCC cell invasion because ATX transcripts and LPA receptor type 1 (LPA1) protein levels were higher in HCC than in normal tissue. They also found that MMP-9 is downstream of LPA1. Moreover, LPA-induced MMP-9 expression with subsequent invasion was abrogated by inhibition of phosphoinositol-3 kinase (PI3K) signaling or dominant or negative mutants of protein kinase C and p38 mitogen-activated protein kinase (MAPK) [163]. Increased MMP-2, -9 and VEGF caused by hypoxia (via ERK1/2) is suppressed with Na^+^/H^+^ exchanger 1 (NHE1) inhibited by 5-(*N*-ethyl-*N*-isopropyl) amiloride [164].

### 7.2. MMPs and TIMPs in NASH-related Carcinogenesis

**Figure 3:**
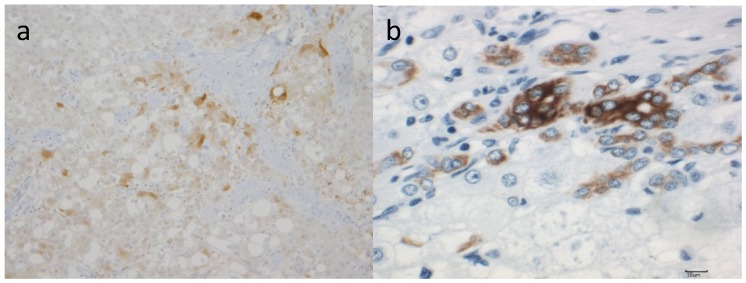
Immunostaining of NASH-derived HCC: (**a**) NASH-derived HCC cells with cytoplasmic reactivity to the MMP-9 antibody are scattered sparsely throughout the nodule (100× magnification); (**b**) NASH-derived HCC cells with cytoplasmic reactivity to the CK19 antibody are also seen (400× magnification).

There are no reports on MMPs and TIMPs in NASH-derived HCC. Figure 3a is a MMP-9 staining microscopic finding of surgically resected NASH-derived HCC. Strong staining is seen in some of the HCC cells diffused in the nodule. MMP-1 and MMP-2 staining are also positive with different distribution (not shown). The tumor size in this case is less than 2 cm in diameter, and the pathological features of HCC cells are well differentiated. According to the hypothesis shown in Figure 2, there is negative staining for both MMP-9 and MMP-2 in small cancer. This discrepancy may be based in the etiology of HCC, because the hypothesis of Figure 2 is based on HBV- or HCV-related HCC while Figure 3a is NASH-related HCC. The involvement of HPCs in NASH appears to be very different from viral chronic liver diseases as discussed above. The present authors are currently investigating the involvement of MMP-positive cells in the dedifferentiation process of early HCC cells in NASH-derived HCC.

MMPs can be influenced by reactive oxygen species (ROS), resulting in the activation of neutrophils and macrophages at the tumor site with inflammation. These oxidants initially activate MMPs via oxidation of the pro-domain cysteine or via modification of amino acids of the catalytic domain by hydrochlorous acid in combination with myeloperoxidase (reviewed in [165]) The methylation status of cytokines in CpG dinucleotides located in the MMP promoter also plays a role in controlling gene expression of MMPs, especially in cancer cells. Recent studies on DNA methylase inhibitors, such as 5-aza-2'deoxycytidine, showed a possible induction of hypomethylation of MMPs genes at the promoter level in human HCC cells [166].

Stefanou *et al.* [167] reported that leptin up-regulated telomerase activity in HepG2 cells through the binding of STAT3 and Myc/Max/Mad network proteins to the promoter of human telomerase reversed transcriptase (hTERT). Leptin could affect the progression and invasion of HCC through its interaction with cytokines and MMPs in the tumorigenic microenvironment. Leptin increased the levels of MMP-13 and MMP-9 in a dose- and time-dependent manner. Insulin receptor-mediated signaling promotes MMP-2 and MMP-9 expression [168]. On the other hand *Kiss*-1 gene, a putative metastasis suppressor gene, is also reported in HCC related with decreased expression of MMP-9 [169].

HPCs derived from bile ducts and the canals in the liver or bone marrow seem to play an important role in the dedifferentiation process from injured hepatocytes ([32], reviewed in [35]). Figure 3b shows the cytoplasmic reactivity to the CK19 antibody in NASH-derived HCC cancer cells. As noted above, one of characteristic pathological findings in NASH is the infiltration of HPCs. Therefore, expression of MMP-1, MMP-2 and MMP-9 seems to be expected in NASH-derived HCC, and the expression is probably different from that found in viral-derived HCC. The regulatory mechanism of TIMPs in NASH-derived HCC also remains unknown [119,170]. Further regulatory research on MMPs and TIMPs should be conducted to prevent the progress of NASH to HCC.

## 8. Conclusions: Future Management of NASH Focusing on MMPs and TIMPs

Distinguishing between benign NAFLD patients and advanced NASH patients is very difficult. To counter this problem the author’s research group has a short-admission program for NASH patients that includes liver biopsy, metabolic examination for glucose tolerance and diet education. We follow up the courses of NASH patients focusing on the complications of HCC. Patients should undergo abdominal echography or CT/MRI and serum markers. Liver tissue obtained by needle biopsy should be checked for stem/progenitor marker-positive cells. Our group is now investigating whether MMP-1 staining or MMP-9 staining is a predictable indicator of HCC. Finally, further investigation of signal transduction pathways in MMPs and TIMPs is essential because future prevention and treatment of NASH-related HCC will be based on gene regulation of MMPs and TIMPs.
